# Neural substrates of individual differences in human fear learning: Evidence from concurrent fMRI, fear-potentiated startle, and US-expectancy data

**DOI:** 10.3758/s13415-012-0089-7

**Published:** 2012-03-27

**Authors:** Sonja van Well, Renée M. Visser, H. Steven Scholte, Merel Kindt

**Affiliations:** 1 University of Amsterdam, Amsterdam, The Netherlands; 2 Department of Clinical Psychology, University of Amsterdam, Roetersstraat 15, 1018 WB Amsterdam, The Netherlands

**Keywords:** Discriminative fear learning, Fear-potentiated startle, fMRI, Amygdala, Hippocampus, Deconvolution, Individual differences, Anxiety

## Abstract

To provide insight into individual differences in fear learning, we examined the emotional and cognitive expressions of discriminative fear conditioning in direct relation to its neural substrates. Contrary to previous behavioral–neural (fMRI) research on fear learning—in which the emotional expression of fear was generally indexed by skin conductance—we used fear-potentiated startle, a more reliable and specific index of fear. While we obtained concurrent fear-potentiated startle, neuroimaging (fMRI), and US-expectancy data, healthy participants underwent a fear-conditioning paradigm in which one of two conditioned stimuli (CS^+^ but not CS^–^) was paired with a shock (unconditioned stimulus [US]). Fear learning was evident from the differential expressions of fear (CS^+^ > CS^–^) at both the behavioral level (startle potentiation and US expectancy) and the neural level (in amygdala, anterior cingulate cortex, hippocampus, and insula). We examined individual differences in discriminative fear conditioning by classifying participants (as conditionable vs. unconditionable) according to whether they showed successful differential startle potentiation. This revealed that the individual differences in the emotional expression of discriminative fear learning (startle potentiation) were reflected in differential amygdala activation, regardless of the cognitive expression of fear learning (CS–US contingency or hippocampal activation). Our study provides the first evidence for the potential of examining startle potentiation in concurrent fMRI research on fear learning.

According to general consensus, emotional memory involves multiple systems that can be distinguished in terms of the information that they process, the ways that they operate, and the brain systems that they depend on (LaBar & Cabeza, 2006; Squire, 2004). Although these independent memory systems generally converge to guide behavior, they may occasionally diverge (see, e.g., LaBar & Cabeza, 2006). Spider-phobic patients, for example, usually know that most spiders are harmless (declarative memory), but the encounter of a spider may nevertheless elicit an autonomic fear response (nondeclarative memory). Human fear conditioning has proven to be an excellent paradigm for the study of the independent but interrelated cognitive (e.g., contingency awareness and US-expectancy ratings) and emotional (e.g., fear-potentiation startle responding) expressions of fear learning (e.g., Hamm & Weike, 2005; Soeter & Kindt, 2010; Weike, Hamm, Schupp, Runge, Schroeder & Kessler, 2005). Indeed, brain lesion and functional magnetic resonance imaging (fMRI) studies have suggested a double dissociation between the cognitive and emotional expressions of fear learning relative to hippocampal and amygdala activation (Bechara, Tranel, Damasio, Adolphs, Rockland & Damasio, 1995; LaBar & Cabeza, 2006; LaBar, LeDoux, Spencer, & Phelps, 1995; Phelps, 2004).

In previous fMRI studies on human fear learning, skin conductance responding (SCR) has been considered to reflect the emotional expression of fear. Most of these studies showed a relationship between conditioned SCR and amygdala activity (e.g., Cheng, Knight, Helmstetter, & Smith, 2006; Cheng, Richards, & Helmstetter, 2007; Knight, Nguyen, & Bandettini, 2005; LaBar, Gatenby, Gore, LeDoux, & Phelps, 1998). There are, however, indications that SCR is a less specific and less reliable index of autonomic fear responding than is startle eyeblink potentiation (measured by electromyography [EMG]). In fact, behavioral studies have suggested that startle potentiation dissociates more strongly from the cognitive expression of fear learning than does SCR (e.g., Hamm & Weike, 2005; Soeter & Kindt, 2010; Weike et al., 2005). In addition, neuroimaging studies have shown that startle potentiation taps directly into the amygdala (Davis, 2006), whereas the amygdala seems to modulate but does not uniquely generate SCR (e.g., Critchley, Melmed, Featherstone, Mathias, & Dolan, 2002; Fredrikson, Furmark, Tillfors Olsson, Fischer, Andersson & Langstrom, 1998; Nagai, Critchley, Featherstone, Trimble, & Dolan, 2004).

Nevertheless, to the best of our knowledge, previous fMRI research on human fear conditioning has never incorporated concurrent measures of EMG startle potentiation as an index of autonomic fear responding. Researchers may have avoided recording EMG startle potentiation during scanning due to technical difficulties. That is, radio frequency pulses and changing magnetic field gradients during imaging induce large artifacts on the EMG signal and, likewise, the EMG equipment may interfere with the quality of the magnetic resonance images. However, with the use of MRI-compatible equipment, the acquisition of electroencephalographic (EEG) or EMG data (i.e., recordings based on similar principles) during scanning seems to induce artifacts on imaging data that are of minimal magnitude (see, e.g., Bonmasser, Hadjikhani, Ives, Hinton, & Belliveau, 2001; Krakow, Allen, Symms, Lemieux, Josephs & Fish, 2000; van Duinen, Zijdewind, Hoogduin, & Maurits, 2005). Furthermore, at present, numerous methods have been proposed and validated that (a) effectively remove fMRI artifacts from simultaneously obtained EMG or EEG recordings (e.g., Allen, Josephs, & Turner, 2000; for a review, see, e.g., Grouiller, Vercueil, Krainik, Segebarth, Kahane & David, 2007; Laufs, Daunizeau, Carmichael, & Kleinschmidt, 2008) or (b) efficiently acquire EMG or EEG recordings during short intervals in-between scans, when the MRI scanner is not collecting data (e.g., Heller, Greischar, Honor, Anderle, & Davidson, 2011; Liu, Dai, Elster, Sahgal, Brown & Yue, 2000). Therefore, we assumed that the concurrent recording of fMRI and EMG startle potentiation is a technical challenge that should be feasible.

In sum, given the surplus value of startle potentiation as an index of autonomic fear learning, we obtained simultaneous startle potentiation (EMG), neuroimaging (fMRI), and US-expectancy data using a differential fear-conditioning paradigm, in which one of two fear-relevant, visual conditioned stimuli (CS^+^ but not CS^–^) is paired with an aversive unconditioned stimulus (US). In this way, we aimed to examine the emotional expression of discriminative fear conditioning in concurrent relation to its cognitive expression and underlying neural networks. First, we tested whether discriminative fear learning would be evident in differential fear-potentiated startle responding, as well as in differential stimulus activation within the amygdala (e.g., Phelps, 2004).

Moreover, a growing body of research has focused on individual differences in human fear learning (for fMRI studies, see, e.g., Dunsmoor, Prince, Murty, Kragel, & LaBar, 2011; Hartley, Fischl, & Phelps, 2011; Indovina, Robbins, Núñez-Elizalde, Dunn, & Bishop, 2011; for genetic studies, see, e.g., Hajcak, Castille, Olvet, Dunning, Roohi & Hatchwell, 2009; Lonsdorf, Weike, Golkar, Schalling, Hamm & Ohman, 2010). We further examined individual differences in human discriminative fear learning and tested whether successful versus unsuccessful discriminative startle potentiation would be reflected, respectively, in normal versus impaired stimulus differentiation within the amygdala.

Furthermore, in human fear-conditioning research, multiple indices of the behavioral expression of fear (US expectancy, SCR, and startle potentiation) are usually obtained for reasons of cross-validation. Although it has long been recognized that independent indices of fear learning do not always converge (e.g., Hodgson & Rachman, 1974), the implications of this divergence for the understanding of pathological fear have never been considered. To provide additional insight into the divergence between emotional and cognitive expressions of fear learning, we examined whether neural substrates (i.e., amygdala and hippocampus) would support our simultaneously obtained behavioral data (i.e., startle potentiation and US expectancy).

## Method

### Participants

We obtained simultaneous EMG, US-expectancy, and fMRI recordings from 40 healthy undergraduate students (14 men, 26 women) between the ages of 18 and 29 years (*M* = 22.4, *SD* = 2.6). All participants were right-handed, had previous experience with fMRI scanning, reported no history of psychiatric or neurological disorders, and reported no current use of any psychoactive medication.

The ethical committee of the University of Amsterdam approved the study protocol. All participants signed informed consent and were paid for their participation.

### Stimuli

#### Conditioned stimuli

To gain ecological validity, we used fear-relevant stimuli. Two pictures of spiders served as the CSs (IAPS numbers 1200 and 1201; Lang, Bradley, & Cuthbert, 2005). Both images depicted (different) spiders and were back-projected for 10 s onto a translucent screen placed at the foot of the scanner table. Participants viewed the images by means of a mirror attached to the head coil. One of the images (CS^+^) was followed by the US (using 75% reinforcement), whereas the other image (CS^–^) was never followed by the US. Assignment of the images as CS^+^ and CS^–^ was counterbalanced.

#### Unconditioned stimulus

The US was a 2-ms aversive electrical stimulation delivered to the participant’s right shinbone. The intensity of the shock was adjusted individually to a level defined by the participant as “uncomfortable, but not painful” (intensity range 8–48 mA, *M* = 23.9, *SD* = 9.6). Delivery of the shock was controlled by a Digitimer DS7A constant-current stimulator (Hertfordshire, U.K.) through a pair of Ag electrodes with a fixed interelectrode distance. Signa electrode gel (Parker Laboratories Inc., Orange, NJ) was applied as an electrolyte. The electrode leads were grounded through a radio frequency filter.

#### Fear-potentiated startle

The conditioned fear response was measured as potentiation of the acoustic startle reflex. The startle probe was an approximately 100-dB, 40-ms burst of broadband noise administered binaurally through headphones (MR-Confon Peltor Optimex, Magdeburg, Germany). The eyeblink component of the acoustic startle response was acquired via EMG measurements of the right orbicularis oculi muscle. Two 7-mm Ag/AgCl electrodes filled with electrolyte gel (Signa, Parker Laboratories Inc., Orange, NJ) were used. One electrode was positioned 1 cm below the pupil, whereas the other electrode was positioned 1 cm below the lateral canthus. To reduce common noise and to maintain electrically identical paths, a third, ground electrode was placed ±3 cm below the orbicularis oculi pars orbitalis on an electrically neutral site. We used copper electrode leads with carbon current-limiting resistors (13 kΩ) serially connected between the lead and electrode to prevent possible warming of the electrodes in the scanner (Lemieux, Allen, Franconi, Symms, & Fish, 1997). The electrode leads were twisted in order to minimize gradient artifacts on the EMG recordings and were connected to a battery-powered MRI-compatible amplifier (SD MRI 64, Mircomed, Italy) placed outside the scanner bore. Via an optical fiber, EMG signals were then transmitted to the recording computer outside the scanner room. The data were sampled at 2048 Hz and recorded using the SystemPLUS software (Micromed, Italy).

**Fig. 1:**
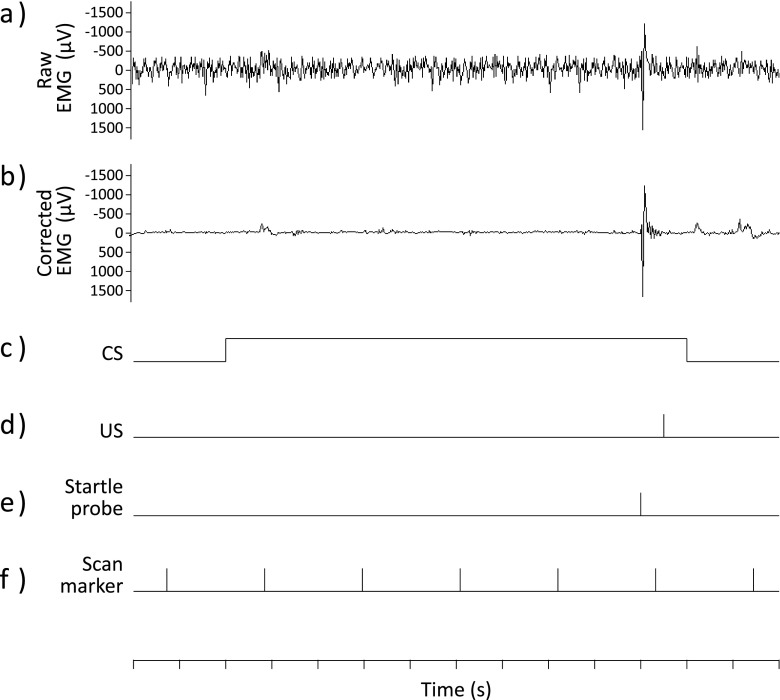
Electromyographic (EMG) traces, along with a schematic representation of the stimulus presentation during scanning. The upper panels show typical EMG recordings before (**a**) and after (**b**) magnetic resonance imaging artifact correction. The lower panels illustrate the presentation of the conditioned stimulus (CS), the unconditioned stimulus (US), and the startle probe (panels **c**, **d** and **e**, respectively) and indicate the start of the acquisition of a brain volume (**f**)

The raw EMG data set was offline corrected for scanner artifacts using the Brain Vision Analyzer software (version 1.05, Brain Products GmbH, Munich, Germany). First, each EMG data file was up-sampled to 20480 Hz and slice volumes were aligned (for synchronizing the clocks of the EMG and fMRI devices). Thereafter, gradient and pulse artifacts were removed, according to the algorithm proposed by Allen et al. (2000), by subtracting an artifact template from the EMG data using a baseline-corrected sliding average of 25 consecutive volumes. Each corrected EMG signal was then down-sampled to the initial sampling frequency (2048 Hz) and low-pass filtered (512 Hz) to reduce residual scanner artifacts. In addition, a 50-Hz notch filter was applied. Visual inspection of the raw and corrected EMG data indicated that the average template subtraction method was effective in removing MRI artifacts from the EMG signal (see Fig. 1a and b). Subsequently, to compute fear-potentiated startle responses, peak amplitudes were identified from the corrected EMG data over the period of 200 ms following startle probe onset by using the home-built VSRRP98 software (VSRRP98: www.test.uva.nl/ozi_psychology/index.php?Page=Software). To adjust for between-participants differences in startle responsivity, startle responses were converted to *t* scores, within participants for each day separately. Finally, missing data (*M* = 5.6%, *SD* = 6.6%) were replaced by using the linear-trend-at-point method.

#### US expectancy

Participants rated the likelihood of US delivery on a 3-point scale anchored with −1 (*no shock*), 0 (*maybe shock*), and 1 (*certainly shock*) using an MRI-compatible response box. They were instructed to rate the US expectancy on each trial and to respond within 5 s after stimulus onset.

#### Neuroimaging

Imaging was conducted using a 3-T MRI scanner (Philips, Intera) with a standard eight-channel head coil. While participants were subjected to the differential fear-conditioning paradigm, whole-brain blood-oxygenation-level-dependent MRI images were acquired (GE-EPI, TR = 2,120 ms, TE = 30 ms, FA = 80º, FOV = 220 mm, matrix = 96 × 96, slice thickness = 3 mm; 37 axial slices sequentially acquired). Additionally, to allow for anatomical localization of functional activation, a T1-weighted anatomical image was obtained for each participant (TR = 9 ms, TE = 3.5 ms, FA = 8º, FOV = 232 × 170 mm, matrix = 256 × 256, slice thickness = 1 mm; 170 coronal slices sequentially acquired).

The imaging data were further processed and analyzed using FSL (FMRIB’s Software Library: www.fmrib.ox.ac.uk/fsl) and MATLAB (The MathWorks, Natick, MA) software. First, functional images were motion corrected (MCFLIRT; Jenkinson, 2002), slice-time corrected (Fourier-space time series phase-shifting), spatially smoothed (5-mm full-width-at-half-maximum Gaussian kernel), and high-pass filtered (cutoff = 100 s). Structural images were brain extracted (BET; Smith, 2002). Subsequently, for each participant, functional images were aligned to the structural image and transformed, on the basis of this structural image, to standard Montreal Neurological Institute (MNI) space using a 12-degree-of-freedom affine registration followed by nonlinear warping. Thereafter, functional MRI data were analyzed using deconvolution analyses (Glover, 1999). For each stimulus type (i.e., CS^+^ or CS^–^), we obtained hemodynamic response functions. To be able to separate neural activation in response to the CS^+^ from activation evoked by the US, the reinforced (75%) as well as the unreinforced (25%) CS^+^ trials were included in the deconvolution analyses. From the obtained hemodynamic time courses, peak activation was determined and the area under the curve (AUC) was calculated across an 8-s window (i.e., 3–10 s after stimulus onset). We used both parameters as indexes of the response magnitude produced by each stimulus type. AUC values might be more sensitive to overall differences in neural activity, whereas the peak values might be more sensitive to differences related to the shape of the hemodynamic response curve.

We restricted our analyses to a set of a-priori-defined regions of interest (ROIs). The amygdala, anterior cingulate cortex (ACC), and insula were selected on the basis of their central role in human fear conditioning (for a review, see Sehlmeyer, Schöning, Zwitserlood, Pfleiderer, Kircher, Arolt & Konrad, 2009), whereas the hippocampus was selected for its recognized role in contingency learning (e.g., Knight, Smith, Cheng, Stein, & Helmstetter, 2004). The amygdala is presumably critically involved in the acquisition and expression of conditioned fear (Kim & Jung, 2006). To allow for possible hemispheric lateralization effects (Baas, Aleman, & Kahn, 2004; Baker & Kim, 2004), we analyzed left and right volumes separately for the amygdala, hippocampus, and insula. We were further interested in the rostral-versus-dorsal division of the ACC, as these two distinct areas have been associated with emotional and cognitive processing, respectively (Bush, Luu, & Posner, 2000). The subcortical ROIs (amygdala and hippocampus) were anatomically defined (left and right) using the Harvard–Oxford subcortical structural atlas (as implemented in FSL). To reduce noise in the cortical ROIs (ACC and insula), we created 16-mm (diameter) spheres centered around peak coordinates based on meta-analytic reviews (for dorsal ACC, *x* = 4, *y* = 14, *z* = 36; for rostral ACC, *x* = −2, *y* = 44, *z* = 20 [Bishop, Duncan, Brett, & Lawrence, 2004]; for left/right anterior insula, *x* = −40/40, *y* = 16, *z* = −6 [Mechias, Etkin, & Kalish, 2010]). All coordinates are reported in MNI standard space. To correct for multiple testing (eight ROIs), we used false discovery rate (FDR ≤ 5%) control (Benjamini & Hochberg, 1995).

#### Subjective assessment

The following subjective assessments on the participant and US characteristics were obtained to explore potential differences between conditionable and unconditionable participants. The Anxiety Sensitivity Index (ASI; Peterson & Reiss, 1992) was used to assess participants’ tendency to respond fearfully to anxiety-related symptoms. Their degrees of spider fear were assessed using the Spider Phobic Questionnaire (SPQ; Klorman, Weerts, Hastings, Melamed, & Lang, 1974). Furthermore, trait anxiety was assessed using the State–Trait Anxiety Inventory (STAI; Spielberger, Gorsuch, & Lushene, 1970). In addition, participants were instructed to rate the US by means of the arousal and valence dimensions of the Self-Assessment Manikin (SAM; Bradley & Lang, 1994) using a 9-point Likert scale.

#### Procedure

Outside the scanner room, the participants first completed the ASI and SPQ. After they had been familiarized with the spider images, they were instructed to look carefully at the images, as one of the images would most of the time be followed by the electrical stimulus, whereas the other image would never be followed by the electrical stimulus. Participants were further instructed to learn to predict the electrical stimulus and to give US-expectancy ratings for each stimulus within 5 s following stimulus onset.

Next, EMG and shock electrodes were attached, the intensity of the shock was set, and the participants were placed in the scanner. Foam pads were placed around participants’ heads to minimize head movement during the scan. For attenuation of scanner noise as well as administration of the startle probes, the participants wore headphones.

Inside the scanner, participants were exposed to a differential fear-conditioning procedure with partial reinforcement. To reduce initial startle reactivity, fear acquisition was preceded by a habituation phase consisting of 10 startle probes presented alone (i.e., noise alone [NA] trials). During fear acquisition, both the CS^+^ and CS^–^ were presented eight times, with 75% of the CS^+^ presentations being reinforced. US onset was delayed 9.5 s after CS^+^ onset and coterminated with the CS^+^ presentation. For each CS, a startle probe was presented 9 s after CS onset (see Fig. 1c–f for a graphical representation of a typical trial). The late onset of the US and startle probe minimized their effect on the neuroimaging data in response to the CS. In addition, eight NA trials were presented. Trial order (i.e., CS^+^, CS^–^, and NA) and intertrial interval (ITI) were quasirandom, such that no more than two consecutive trials or ITIs were of the same type. The ITIs varied between 15, 20, and 25 s (*M* = 20 s).

Outside the scanner room, participants completed the STAI-T and evaluated the US. Furthermore, to conclude the experiment, the participants were asked to indicate which image was followed by the shock during a postexperimental interview, in order to assess participants’ awareness of the CS–US contingency.

## Results

As a result of proper participant safety precautions, no adverse events took place during the concurrent acquisition of the startle potentiation, fMRI, and US-expectancy data. More specifically, none of the participants reported any warming of the EMG electrodes during scanning. The data from one participant were excluded from the EMG analyses due to incomplete EMG data acquisition (>25% missing values). In addition, the data of three participants were removed from the fMRI analyses due to recording problems (*n* = 1) or excessive head movements, defined as any sudden head movements exceeding half a voxel size (i.e., 1.5 mm; *n* = 2). All of the hypotheses were tested one-tailed with a significance level of *p* < .05. Any remaining differences were tested two-tailed (and are labeled as such in the text).

### Fear-potentiated startle

To examine fear acquisition, we compared differential fear-potentiated startle responding (CS^+^ vs. CS^–^) during early acquisition (Trials 1–2) with that during late acquisition (Trials 7–8). Analyses of variance showed successful fear acquisition by a significant increase in the differential startle response (CS^+^ vs. CS^–^) from early to late acquisition [Stimulus × Trial: *F*(1, 38) = 13.12, *p* < .001, *η*_p_^2^ = .26]. Indeed, the fear-conditioned stimulus (CS^+^) elicited significantly more startle potentiation than did the control stimulus (CS^–^) during late acquisition [*t*(38) = 6.39, *p* < .001, *d* = 1.03], but not during early acquisition [*t*(38) < 1.0].

Subsequently, we classified participants according to whether they showed successful differential startle responding over the course of fear learning. Those participants who showed stronger fear-potentiated startle responses to the CS^+^ than to the CS^–^ during the second half of acquisition and for whom the differentiation between the CSs was stronger during the second than during the first half of acquisition were classified as conditionable (*n* = 24). The remaining participants (*n* = 15) were classified as unconditionable.

**Fig. 2:**
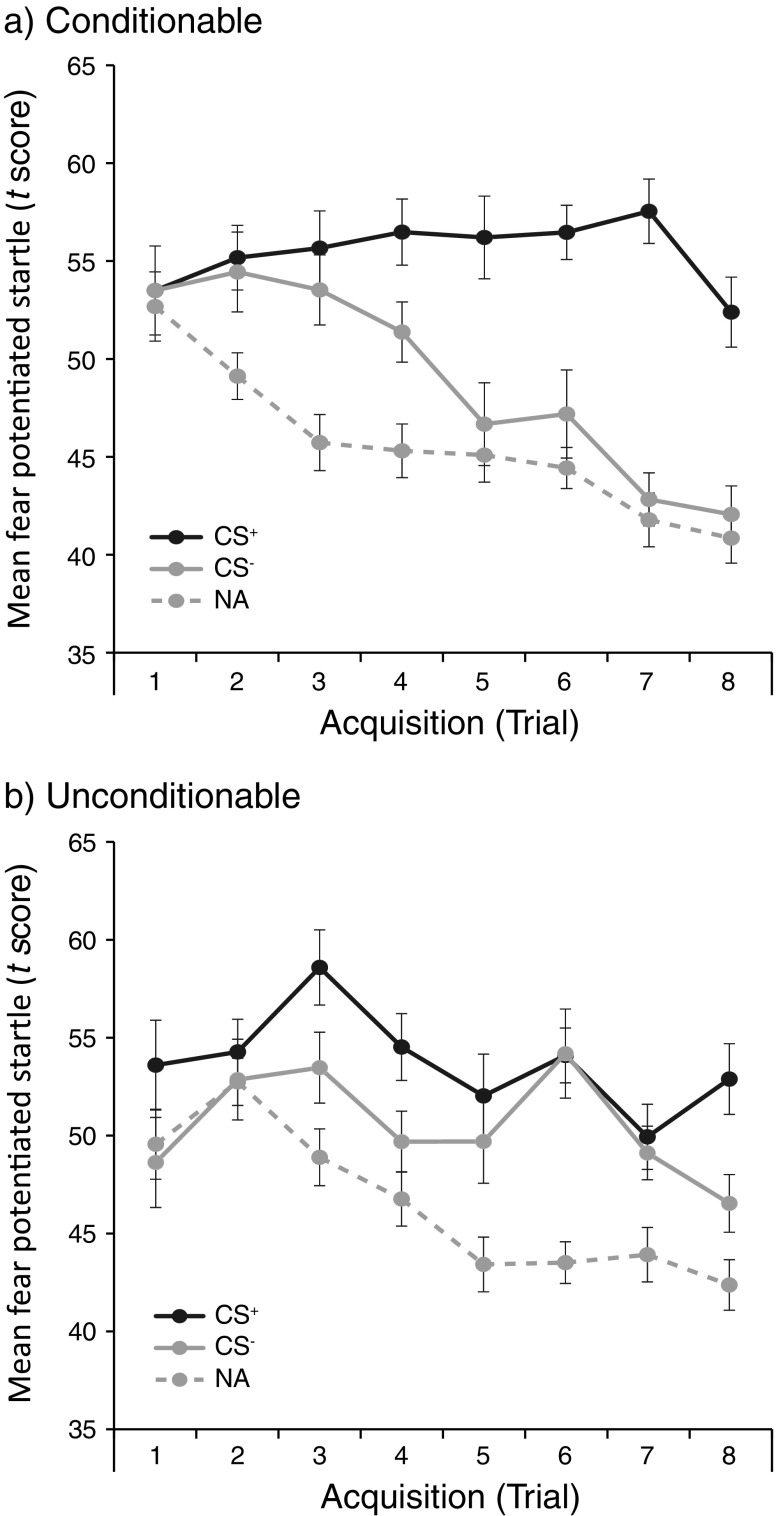
Startle potentiation in mean fear-potentiated startle responses (*t* score) to the fear-conditioned stimulus (CS^+^), the control stimulus (CS^–^), and noise alone (NA) trials for the (**a**) conditionable (*n* = 24) and (**b**) unconditionable (*n* = 15) participants. Error bars depict standard errors of the means

As a result of our classification, the conditionable participants differed from the unconditionable participants in fear acquisition [Stimulus × Trial × Group: *F*(1, 37) = 8.92, *p* < .01, *η*_p_^2^ = .19; see Fig. 2]. That is, the conditionable participants showed successful fear acquisition, as indicated by a significant increase in differential startle potentiation (CS^+^ vs. CS^–^) from early (Trials 1–2) to late (Trials 7–8) acquisition [Stimulus × Trial: *F*(1, 23) = 31.52, *p* < .001, *η*_p_^2^ = .58], whereas the unconditionable participants showed no fear acquisition, as indicated by a lack of increased differential startle potentiation over the course of training [Stimulus × Trial: *F*(1, 14) < 1.0].

Interestingly, the lack of discriminative fear learning in the unconditionable participants did not result from a reduction in startle potentiation to the fear-conditioned stimulus [CS^+^; Trial × Group: *F*(1, 37) < 1.0], but rather from prolonged startle responding to the control stimulus [CS^–^; Trial × Group: *F*(1, 37) = 6.96, *p* < .05, *η*_p_^2^ = .16], as compared to the conditionable participants. More specifically, in contrast to the conditionable participants, who showed a reduction of startle potentiation from early to late acquisition to the CS^–^ [*t*(23) = 5.29, *p* < .001, *d* = 1.09], the unconditionable participants did not show such a reduction in startle responding [*t*(14) < 1.3]. In fact, during late acquisition, the unconditionable participants showed a higher level of startle potentiation in response to the CS^–^ than did their conditionable counterparts [*t*(37) = 2.94, *p* < .01, *d* = 1.00; see Fig. 2].

### Descriptives

In Table 1, we present the participant and US characteristics for the conditionable and unconditionable participants, separately. Relative to the conditionable group, participants in the unconditionable group reported significantly higher levels of anxiety sensitivity [*t*(37) = 2.23, *p* < .05, two-tailed, *d* = 0.70; see Table 1]. The conditionable and unconditionable participants did not differ in terms of trait anxiety, spider fear, self-selected US intensity level, or US evaluation [all *t*s(37) < 1.0].

**Table 1:** Summary of participant and US characteristics by group

	Conditionable (*n* = 24)		Unconditionable (*n* = 15)	
	*M*	*SD*	*M*	*SD*
ASI	7.9	3.8	11.1	5.3^*^
STAI-T	35.1	7.5	35.9	8.7
SPQ	4.9	5.7	5.0	6.0
US intensity (mA)	24.7	8.9	23.1	11.0
US evaluation				
SAM Valence	6.8	0.9	6.9	0.8
SAM Arousal	3.9	1.3	3.9	1.2

ASI, Anxiety Sensitivity Index; STAI-T, State–Trait Anxiety Inventory: Trait scale; SPQ, Spider Phobia Questionnaire; US, unconditioned stimulus; SAM, Self-Assessment Manikin. ^*^ *p <* .05.

### US expectancy

**Fig. 3:**
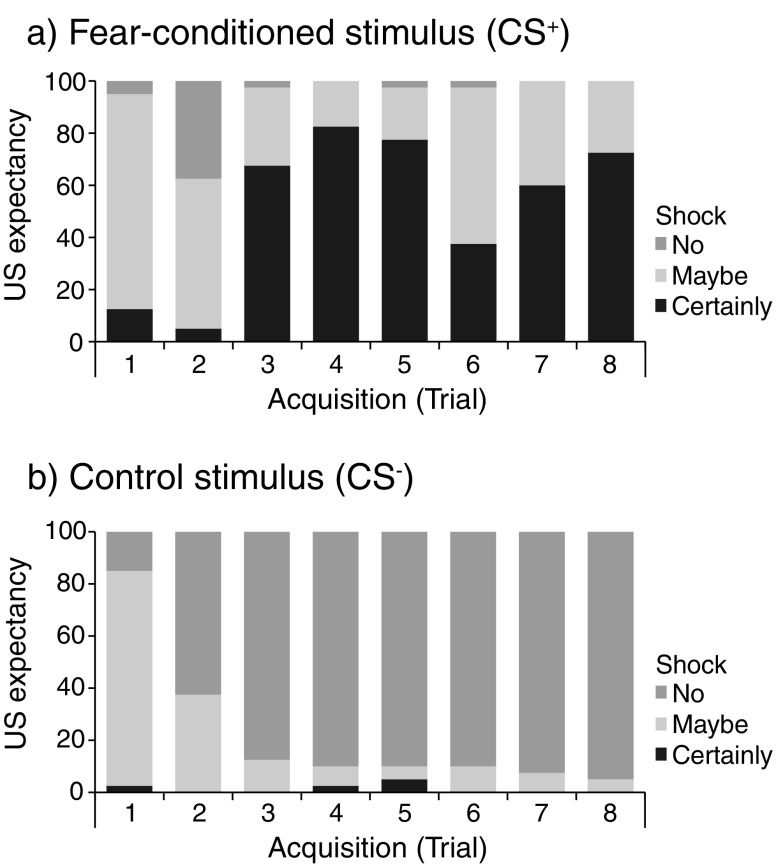
US-expectancy ratings: Percentages of responses in each of the three response categories (i.e., *certainly shock*, *maybe*, and *no shock*) for the (**a**) fear-conditioned (CS^+^) and (**b**) control (CS^–^) stimuli

Figure 3 shows the US-expectancy data over the course of fear acquisition for both CSs separately. To analyze learning-related changes in CS–US contingency, the ordinal US-expectancy ratings were collapsed across the CS^+^ and CS^–^ into three categories: (1) aware of CS–US association (i.e., CS^+^ = certainly shock and CS^–^ = no shock), (2) uncertain CS–US association (i.e., CS^+^ = certainly shock and CS^–^ = maybe shock, or CS^+^ = maybe shock and CS^–^ = no shock), and (3) not aware of CS–US association (i.e., all remaining combinations of CS–US expectancies). Given that our ordinal data do not allow us to calculate the means over the first two and the last two acquisition trials (cf. startle data), we analyzed early versus late acquisition by comparing Trial 1 with Trial 8. As expected, the results of Friedman’s test revealed a significant change in participants’ CS–US expectancy between early and late acquisition [*χ* ^2^(1, *N* = 40) = 34.0, *p* < .001, *W* = .85].

Contrary to the fear-potentiated startle responses, Friedman’s test on US-expectancy ratings revealed learning-related changes not only for the conditionable group [*χ* ^2^(1, *N* = 24) = 21.0, *p* < .001, *W* = .88], but also for the unconditionable group [*χ* ^2^(1, *N* = 15) = 12.0, *p* < .005, *W* = .80]. The majority of participants, in both the conditionable and the unconditionable groups, were unaware (87.5% and 60.0%, respectively) or uncertain (12.5% and 33.3%, respectively) about the CS–US association at early acquisition, but had learned the CS–US association at late acquisition (66.7% and 80.0%, respectively). Furthermore, a Mann–Whitney *U* test revealed that the US-expectancy ratings of the conditionable participants did not differ from those of the unconditionable participants at late acquisition (*z < −*1.0). In addition, all participants were aware of the CS–US contingency, as assessed by means of the postexperimental interview.

### Neuroimaging

**Fig. 4:**
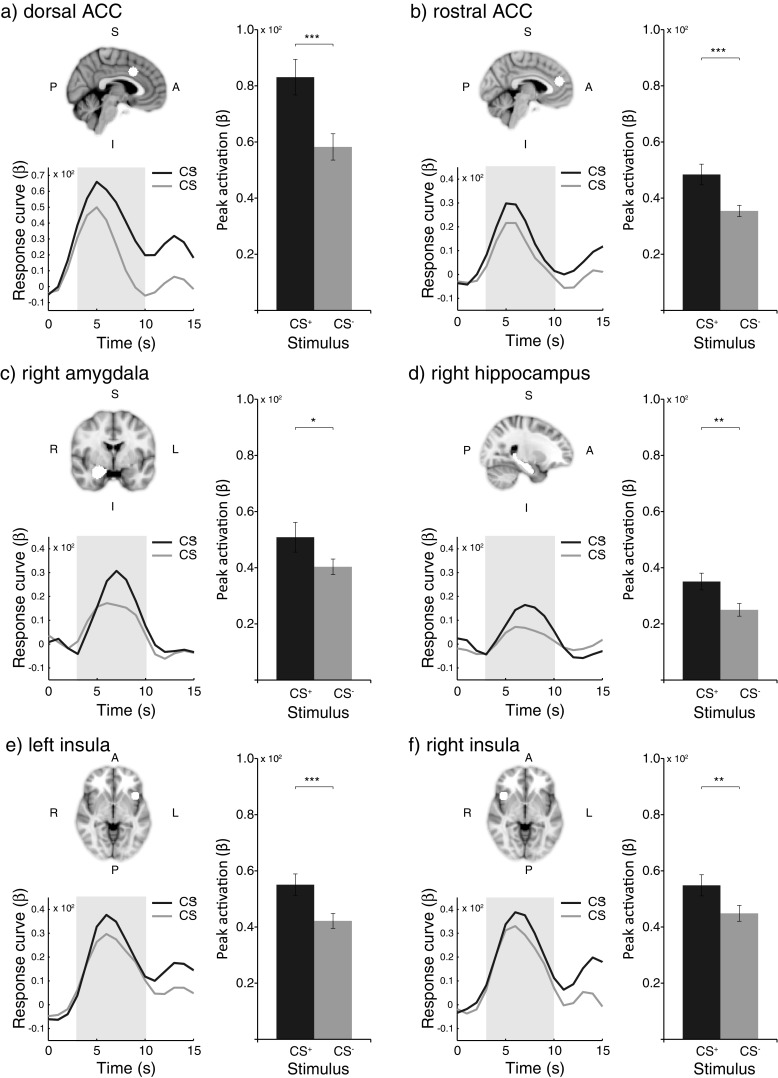
Summary of the main neuroimaging findings in response to the fear-conditioned (CS^+^) and control (CS^–^) stimuli within the (**a**) dorsal anterior cingulate cortex (dorsal ACC; *x* = 4, *y* = 14, *z* = 36), (**b**) rostral anterior cingulate cortex (rostral ACC; *x* = −2, *y* = 44, *z* = 20), (**c**) right amygdala (*x* = 24, *y* = −2, *z* = −20), (**d**) right hippocampus (*x* = 28, *y* = −22, *z* = −14), (**e**) left insula (*x* = −40, *y* = 16, *z* = −6), and (**f**) right insula (*x* = 40, *y* = 16, *z* = −6). The coordinates are reported in MNI standard space. The upper left image in each panel shows the ROI location; the bottom left graph in each panel depicts the time courses of the hemodynamic responses (area under the hemodynamic response curve [AUC] calculated from 3 to 10 s after stimulus onset, as indicated by the gray area). The right graph in each panel shows peak activation. A, anterior; I, inferior; L, left; P, posterior; R, right; S, superior. ^***^ *p <* .001, false discovery rate (FDR) corrected. ^**^ *p <* .01, FDR corrected. ^*^ *p <* .05, FDR corrected

To examine discriminative neural activation in response to the fear-conditioned and control stimuli, we used deconvolution analyses. For each stimulus type, we obtained mean hemodynamic response curves over the course of fear learning (Trials 1–8). A summary of the main neuroimaging findings is presented in Fig. 4. This figure depicts ROI locations as well as hemodynamic response curves and peak responses to the CS^+^ and CS^–^ for the ACC (dorsal and rostral), right amygdala, right hippocampus, and insula (left and right). As can be seen, the response curves we obtained (see Fig. 4) resemble typical fMRI hemodynamic response time courses.

### CS activation

#### Peak

In parallel with the behavioral data, the neuroimaging data showed successful fear conditioning, as indicated by overall differential peak activation (CS^+^ vs. CS^–^; see Fig. 4). All ROIs, except for the left hippocampus [ts(36) < 1.0], revealed significantly higher peak values in the hemodynamic response curves evoked by the CS^+^, as opposed to the CS^–^ [ts(36) = 1.90 to 4.27, ps < .05, d = 0.33 to 0.72; all effects survived FDR correction], with a trend significant effect for the left amygdala [t(36) = 1.63, p = .056, d = 0.28].

#### Mean (AUC)

Similar to the peak effects, analyses on the mean AUCs revealed that the CS^+^ elicited more activation than did the CS^–^ within the dorsal ACC [*t*(36) = 3.63, *p* < .005, *d* = 0.64, FDR corrected]. The rostral ACC revealed a similar trend significant effect [*t*(36) = 1.47, *p* = .076, *d* = 0.25]. For the remaining ROIs, we found a lateralized pattern of differential AUC stimulus activation. Paired-samples *t* tests revealed significantly more activation to the CS^+^ than to the CS^–^ within the right hippocampus [*t*(36) = 2.15, *p* < .05, *d* = 0.38], and trends were found in the right amygdala and right insula [*t*(36) = 1.48, *p* = .074, *d* = 0.26, and *t*(36) = 1.49, *p* = .073, *d* = 0.25, respectively], but not within the left hippocampus, left amygdala, or left insula [all *t*s(36) < 1.2]. However, none of the right-sided effects survived FDR correction.

### CS activation for the conditionable versus unconditionable participants

#### Peak

Analyses of variance on CS peak activation showed a significant difference in stimulus differentiation for the conditionable participants relative to the unconditionable participants within the right amygdala [Stimulus × Group: *F*(1, 34) = 5.59, *p* < .05, *η*_p_^2^ = .14, FDR corrected] and showed a similar trend in the left amygdala [Stimulus × Group: *F*(1, 34) = 2.18, *p* = .075, *η*_p_^2^ = .06]. Moreover, as expected, this group differentiation did not emerge for hippocampal activation [both left and right; Stimulus × Group: *F*s(1, 34) < 1.0].

**Fig. 5:**
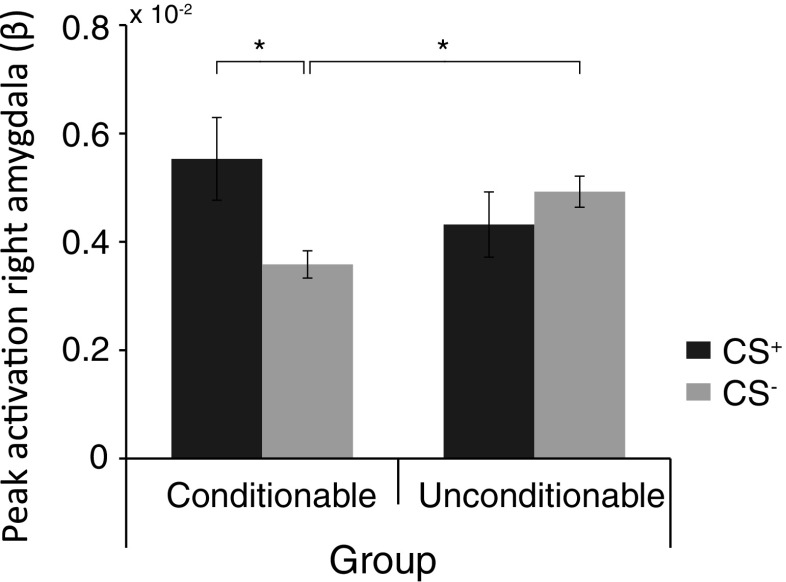
Peak activations for the right amygdala in response to the fear-conditioned (CS^+^) and control (CS^–^) stimuli for the conditionable and unconditionable participants, separately. ^*^ *p <* .05

Follow-up *t* tests revealed that the conditionable participants differentiated between CS^+^ and CS^–^ presentation within the right and left amygdala [CS^+^ > CS^–^: *t*(21) = 2.82, *p* < .01, *d* = 0.77, and *t*(21) = 1.87, *p* < .05, *d* = 0.44, respectively], whereas the unconditionable participants did not [both *t*s(13) < 1.0]. Moreover, the conditionable and unconditionable participants showed similar levels of right amygdala peak activation on the CS^+^ [*t*(34) < 1.1], but differed on the CS^–^ [*t*(34) = 2.56, *p* < .05, two-tailed, *d* = 0.85]. In fact, the CS^–^, the stimulus that should signal a period of safety, evoked higher right amygdala peak responding for the unconditionable participants than for the conditionable participants (see Fig. 5).

Furthermore, we found a similar Stimulus × Group interaction for the dorsal ACC [*F*(1, 34) = 6.34, *p* < .05, *η*_p_^2^ = .16, FDR corrected], but not for the rostral ACC [*F*(1, 34) < 1.0]. Additionally, trend significant Stimulus × Group interactions emerged for the insula [*F*(1, 34) = 2.52, *p* = .061, *η*_p_^2^ = .07, and *F*(1, 34) = 2.00, *p* = .083, *η*_p_^2^ = .06, for the left and right insula, respectively]. As with the amygdala, follow-up analyses revealed that the conditionable participants showed differential stimulus peak activation (CS^+^ > CS^–^) within the dorsal ACC [*t*(21) = 5.61, *p* < .001, *d* = 1.33] and the insula [*t*(21) = 3.66, *p* < .001, *d* = 0.86, and *t*(21) = 3.87, *p* < .001, *d* = 0.87, for left and right insula, respectively], whereas the unconditionable participants did not [all *t*s(13) < 1.0].

#### Mean (AUC)

We observed no significant Stimulus × Group interaction for mean CS activation within the amygdala (left/right, *F*s < 1.3) or the remaining ROIs (all *F*s < 1.0).

### US activation

One might reason that the unconditionable participants lacked differential fear conditioning because they were not—or at least, were less emotionally—engaged in the fear-conditioning paradigm than were their conditionable counterparts. We already reported that the unconditionable participants did not show reduced differential startle fear responding and amygdala activation to the conditioned stimulus (CS^+^) but showed enhanced responding to the control stimulus (CS^–^) instead. Also, note that the conditionable and unconditionable participants did not differ in self-selected US intensity or their US evaluations (see Table 1). These findings argue against a lack of emotional engagement. In addition, we evaluated group differences in amygdala activation to the US. Interestingly, for the right amygdala, independent *t* tests revealed that the unconditionable participants (*M*_Peak_ = 0.73 × 10^-2^, *SD*_Peak_ = 0.35 × 10^-2^, and *M*_AUC_ = 0.32 × 10^-2^, *SD*_AUC_ = 0.33 × 10^-2^) showed stronger activation in response to the US than did the conditionable participants (*M*_Peak_ = 0.52 × 10^-2^, *SD*_Peak_ = 0.26 × 10^-2^, and *M*_AUC_ = 0.17 × 10^-2^, *SD*_AUC_ = 0.22 × 10^-2^) [*t*_Peak_(34) = 2.13, *p* < .05, two-tailed, *d* = 0.69, and *t*_AUC_(34) = 1.71, *p* = .097, two-tailed, *d* = 0.55]. An additional Spearman’s correlation analysis revealed that US peak activation correlated negatively with differential CS peak activation within the right amygdala [*r*(36) = −.33, *p* < .05]. Specifically, a higher level of right amygdala activation in response to the US was associated with less differentiation between the CS^+^ and CS^–^ within the same brain area. Taken together, our findings suggest that heightened amygdala responding to the aversive stimulus (US) hampered discriminative fear conditioning in the unconditionable participants.

This remarkable finding on US activation should be interpreted with caution, given that the electrical shock may have produced motion artifacts. Nonetheless, participants with excessive head movement were removed from the analyses, and additional analyses on the mean relative displacement that was used for motion correction (MCFLIRT; Jenkinson, 2002) revealed no group difference (*t* < 1.0; *M* = 0.074 mm, *SD* = 0.014 mm).

### Association between startle potentiation and neural activation

**Fig. 6:**
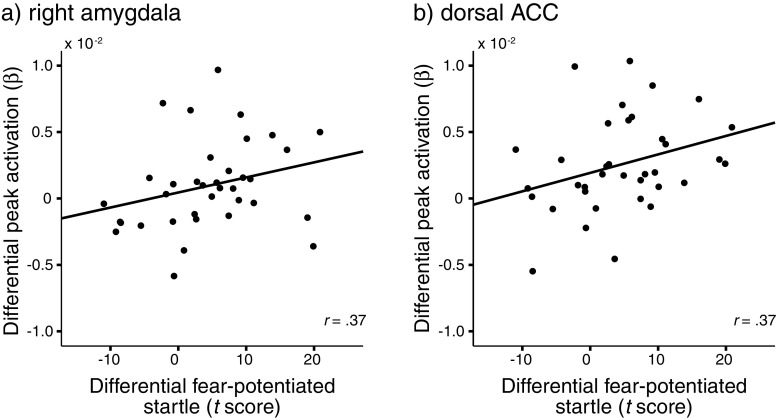
Correlations between autonomic fear responding and neural activation. The panels show the correlation between differential startle potentiation $\left( {{\text{C}}{{\text{S}}^{ + }}_{{{\text{Trial}}5-8}}-{\text{C}}{{\text{S}}^{-}}_{{{\text{Trial}}5-8}}} \right)-\left( {{\text{C}}{{\text{S}}^{ + }}_{{{\text{Trial}}1-4}}-C{S^{-}}_{{{\text{Trial}}1-4}}} \right)$ and differential neural activation $\left( {{\text{C}}{{\text{S}}^{ + }}_{{{\text{Trial}}1-8}}-C{S^{-}}_{{{\text{Trial}}1-8}}} \right)$ for the (**a**) right amygdala and (**b**) dorsal ACC, anterior cingulate cortex; CS, conditioned stimulus

To relate automatic behavioral fear responding to the underlying neural substrates, we calculated Spearman’s correlation coefficients between the differential startle response $\left[ {\left( {{\text{C}}{{\text{S}}^{ + }}_{{{\text{Trials}} \ 5-8}}-{\text{C}}{{\text{S}}^{-}}_{{{\text{Trials}} \ 5-8}}} \right)-\left( {{\text{C}}{{\text{S}}^{ + }}_{{{\text{Trials}} \ 1-4}}-{\text{ C}}{{\text{S}}^{-}}_{{{\text{Trials}} \ 1-4}}} \right)} \right]$ and the differential neural activation $\left( {{\text{C}}{{\text{S}}^{ + }}_{{{\text{Trials}}1-8}}-{\text{C}}{{\text{S}}^{-}}_{{{\text{Trials}}1-8}}} \right)$.^1^ These analyses revealed a significant correlation between the emotional expression of fear and neural activation within the right amygdala [*r*(36) = .37, *p* < .05], as well as within the dorsal ACC [*r*(36) = .37, *p* < .05; see Fig. 6a and b].

## Discussion

We designed the present study to examine individual differences in the emotional expression of discriminative fear learning (as indexed by EMG startle potentiation) in direct relation to its more cognitive expression (US expectancy) and underlying neural substrates. In line with previous research, discriminative fear learning was evident from the differential expression of fear (CS^+^ > CS^–^), both at the behavioral level (startle potentiation and US expectancy) and at the neural level (ACC, amygdala, hippocampus, and insula). Moreover, we examined individual differences in discriminative fear learning by classifying participants according to whether they showed successful fear conditioning, as indicated by differential startle potentiation. Here we demonstrated that the conditionable participants were aware of the stimulus contingency and revealed stimulus differentiation within the amygdala and hippocampus. The unconditionable participants, on the other hand, showed impaired stimulus differentiation within the amygdala, while leaving the more cognitive expression of fear learning intact, as indicated by US-expectancy ratings and hippocampal activation. Interestingly, our findings suggest that relatively heightened amygdala activation in response to the US hampered discriminative fear conditioning in the unconditionable participants.

Discriminative fear learning, however, is crucial and adaptive, in that it allows us to predict and avoid future dangers. The rapid or exaggerated acquisition of aversive associations (“hyperconditionability”; Eysenck, 1979; Orr, Metzger, Lasko, Macklin, Peri & Pitman, 2000), as well as the inability to inhibit fear responses in the presence of safety (impaired safety signal learning; Davis, Falls, & Gewirtz, 2000; Jovanovic, Kazama, Bachevalier, & Davis, 2012), may be maladaptive and associated with the development of anxiety disorders (Lissek, Powers, McClure, Phelps, Woldehawariat, Grillon & Pine, 2005; Mineka & Oehlberg, 2008). The findings of our study are in line with the impaired safety signal learning perspective (Davis, Falls & Gewirtz, 2000). As opposed to the conditionable participants, who showed a reduction of startle potentiation from early to late acquisition to the stimulus that signaled a period of safety, the unconditionable participants did not reveal such a reduction of fear-potentiated startle responding. Furthermore, in response to the control stimulus, the unconditionable participants showed higher levels of startle potentiation and right amygdala activation as compared to the conditionable participants. In addition to lesion studies (Bechara et al., 1995; LaBar, LeDoux, Spencer & Phelps, 1995), in which amygdala-damaged patients have shown impaired fear conditioning due to amygdala inactivation, the results of our study suggest that relatively heightened amygdala activation might have impaired discriminative fear learning in the unconditionable participants. Actually, although they are speculative, our findings suggest that the heightened activation of the right amygdala in response to the shock in the unconditionable participants might have hampered the forming of a *specific* aversive association between the fear-conditioned stimulus (as opposed to the control stimulus) and the shock. The heightened amygdala response in unconditionable participants could be indicative of increased fear intensity that facilitated the generalization of fear to the safety stimulus (see also Dunsmoor, Mitroff, & LaBar, 2009; Laxmi, Stork, & Pape, 2003). As a result, the unconditionable participants responded equally to both stimuli, even though they noticed the difference in contingency. This autonomic “better safe than sorry” strategy might cause undue fear to safety stimuli that bear a (high) resemblance to the threat stimulus.

The findings of the present study further revealed a higher level of anxiety sensitivity (i.e., fear of anxiety-related sensations) for the unconditionable than for the conditionable participants. This effect of conditionability was specific to the ASI and did not hold for the other measures (i.e., for trait anxiety and fear of spiders). As the ASI indexes fear of anxiety-related bodily sensations, the specific relation between fear conditioning and the ASI could be explained by the physical nature of the threat stimulus (i.e., a shock delivered to the leg). Previous research has not only demonstrated that anxiety sensitivity is related to increased risk of panic attacks (McNally, 2002; Zvolensky & Schmidt, 2007) but has also suggested that the degree of anxiety sensitivity is associated with the severity of post-traumatic stress disorder symptoms (Fedoroff, Taylor, Asmundson, & Koch, 2000). Our finding that the unconditionable participants reported higher anxiety sensitivity than did their conditionable counterparts may therefore indicate a mechanism by which individual differences might result in the overgeneralization of fear, which is proposed to be a core feature in anxiety disorders (e.g., Lissek, Rabin, McDowell, Dvir, Bradford, Geraci & Grillon, 2009; Mineka & Zinbarg, 1996).

Although the present study has not provided a direct comparison between startle potentiation and SCR, the results of our study provide evidence that startle potentiation is a usable and valuable index of the autonomic expression of fear in concurrent neuroimaging research on fear learning and memory. After applying imaging-artifact correction on the raw EMG signal (Allen et al., 2000), we found, in line with previous research (Hamm & Weike, 2005; Kindt, Soeter, & Vervliet, 2009; Soeter & Kindt, 2010, 2011; Weike et al., 2005), that conditioning of EMG startle potentiation dissociated from the more cognitive expression of fear conditioning. Furthermore, by analyzing the concurrent startle potentiation and neuroimaging data, we demonstrated a significant correlation between differential neural activation within the right amygdala and differential autonomic fear responding, as indexed by startle eyeblink potentiation. These findings support the central role of the amygdala in the autonomic expression of fear (Davis, 2006; LeDoux, 2000).

The results of the present study further support roles for the ACC and insula in fear conditioning (cf. Mechias et al., 2010; Sehlmeyer et al., 2009). Furthermore, inconsistent with the functional specialization of dorsal and ventral regions of the ACC (i.e., cognitive vs. emotional processing, respectively; see, e.g., Bush et al., 2000), the results of the present study showed stimulus differentiation for the conditionable participants relative to the unconditionable participants within the dorsal ACC, but not within the rostral ACC. This finding is surprising, given that the dorsal ACC is the putative cognitive region and that the two groups did not differ on the more cognitive expression of fear learning. On the other hand, this finding is in line with accumulating evidence suggesting that the functional specialization of dorsal and rostral regions of the ACC does not hold for fear conditioning. A recent meta-analysis, regarding the neuroimaging data on the role of ACC in the processing of anxiety and fear, concluded that, contrary to the traditional dichotomy, both subdivisions of the ACC make key contributions to emotional processing (Etkin, Egner, & Kalish, 2011). More specifically, this meta-analysis provides strong evidence that the dorsal ACC is involved in the expression of conditioned fear responses (as indexed by SCR).

Some of our neural effects were reported for peak activity values, but not for mean activity (AUC) values. For instance, we observed differences in amygdala and dorsal ACC activation between conditionable and unconditionable participants in the peak analyses, but not in the AUC analyses. This might indicate that the shape of the hemodynamic response curve differs between conditionable and unconditionable participants, but the overall magnitude of the neural response does not, or does so to a lesser degree. These differences could reflect strong but brief activation in response to the CS^+^ for conditionable participants, as opposed to weaker but more sustained activation for unconditionable participants. Yet the consequences of these differences in brain activity—as well as whether it is meaningful that one measure of brain activity detected differences, whereas the other did not—await further investigation.

Some limitations of this study should be noted. First, on the basis of fear-potentiated startle responding, 38% of the participants were classified as unconditionable. As all of the participants were aware of the CS–US contingency, the relatively high proportion of unconditionable participants might raise questions about the reliability of using startle potentiation as an index of autonomic fear conditioning in the scanner. However, further analyses revealed that the unconditionable participants were classified as such not because they were nonresponsive, but because they responded to both stimuli and did not learn to discriminate between the fear-conditioned and the control stimulus. Our fear-conditioning paradigm inside the scanner—which encompassed scanner noise, aversive shocks, and loud startle probes—possibly induced more anxiety and resulted in more generalization of learned fear than would comparable fear-conditioning paradigms performed outside the scanner. We did not repeat the experiment outside the scanner to test this assumption and to cross-validate the use of EMG startle potentiation during scanning. Second, due to methodological variations in data acquisition (e.g., continuous fMRI and EMG recordings vs. ordinal expectancy ratings) and in data processing (e.g., the possibility of separating early vs. late acquisition in the behavioral but not in the neural data) across the different measures of fear learning, we inevitably had to average and analyze over different numbers of trials. This might have reduced to some extent the convergence between the measures that we used.

Taken together, the results reported in the present study provide first evidence for the potential of examining startle potentiation as an index of the emotional expression of fear in fMRI research. The concurrent use of neural (fMRI) and behavioral (both emotional and cognitive) data in fear conditioning may open new ways to investigate individual differences in human fear learning. Research on individual differences regarding the divergence between emotional and cognitive expressions of fear learning and its underlying neural substrates may, for example, provide valuable insights for understanding the transition from adaptive to maladaptive fear learning.

## Footnotes

1 With regard to the deconvolution analyses, the number of unreinforced CS^+^ trials (*n* = 2) in the present research design was enough to separate neural activation in response to the reinforced CS^+^ trials from neural activation in response to the shock (see Fig. 4 for the time courses of the hemodynamic responses). Nevertheless, this low number of unreinforced CS^+^ trials did not allow for separating neural activation into early and late acquisition phases. Therefore, we calculated differential neural activation across all trials instead (CS^+^_Trials 1–8_ – CS^–^_Trials 1–8_).
